# Color-conversion displays: current status and future outlook

**DOI:** 10.1038/s41377-024-01618-8

**Published:** 2024-11-01

**Authors:** Guijun Li, Man-Chun Tseng, Yu Chen, Fion Sze-Yan Yeung, Hangyu He, Yuechu Cheng, Junhu Cai, Enguo Chen, Hoi-Sing Kwok

**Affiliations:** 1 Key Laboratory of Optoelectronic Devices and Systems of Ministry of Education and Guangdong Province, College of Physics and Optoelectronic Engineering, Shenzhen University, Shenzhen, 518060 China; 2 State Key Laboratory of Advanced Displays and Optoelectronics Technologies, Department of Electronic and Computer Engineering, The Hong Kong University of Science and Technology, Hong Kong, China; 3 National and Local United Engineering Laboratory of Flat Panel Display Technology, College of Physics and Information Engineering, Fuzhou University, College of Physics and Information Engineering 350108, Fuzhou, China; 4 Fujian Science & Technology Innovation Laboratory for Optoelectronic Information of China, Fuzhou, 350108 China

**Keywords:** Displays, Quantum dots

## Abstract

The growing focus on enhancing color quality in liquid crystal displays (LCDs) and organic light-emitting diodes (OLEDs) has spurred significant advancements in color-conversion materials. Furthermore, color conversion is also important for the development and commercialization of Micro-LEDs. This article provides a comprehensive review of different types of color conversion methods as well as different types of color conversion materials. We summarize the current status of patterning process, and discuss key strategies to enhance display performance. Finally, we speculate on the future prospects and roles that color conversion will play in ultra-high-definition micro- and projection displays.

## Abstract (web summary)

This article provides a comprehensive review of the current status and future outlook for color-conversion display, focusing on the types of color conversion, color conversion materials, patterning process, and performance improvement strategies.

## Graphical abstract

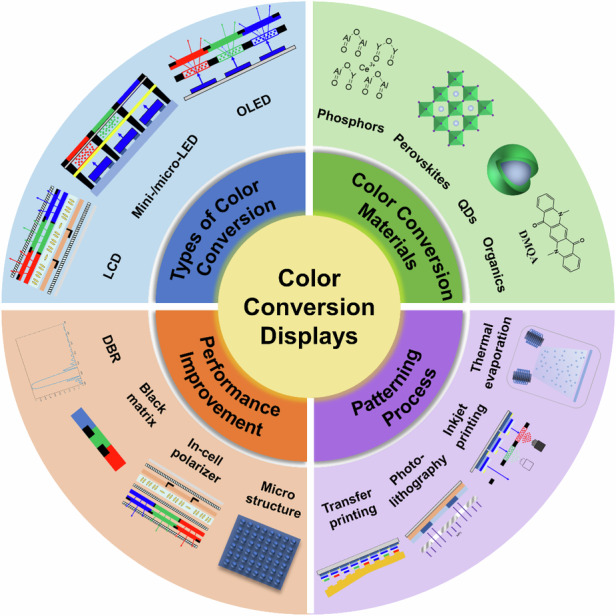

## Introduction

Display technology has become prominent and ubiquitous in our daily life, with widespread applications in augmented reality(AR)/virtual reality(VR) devices, smartphones, tablets, monitors, TVs, etc. At present, liquid crystal displays (LCDs) and organic light-emitting diodes (OLEDs) are two mainstream products in global display industry. Other emerging areas of focus in display include Micro-LED displays, quantum dots (QDs)/perovskite light-emitting displays, and 3D displays, just to name a few^1^. Looking ahead, upcoming technologies such as interactive displays, holographic displays, neuron displays, and light field perception displays are expected to create new application scenarios to meet the increasingly sophisticated visual demands of users^2–5^.

In displays, the increasing demand for high-quality color representation is closely tied to the rising visual expectations of users^6,7^. Color presentation in displays is generally reproduced by mixing red, green, and blue color primaries through two ways: RGB tri-color independent luminescence, and the utilization of a blue excitation light source combined with a color conversion process^8^. The RGB tri-color approach involves using luminescent semiconductors that emit distinct wavelengths corresponding to red, green, and blue emissions^9^. However, achieving comparable quantum efficiencies for RGB colors across different materials is challenging due to variations in electrical and thermal properties, as well as issues like material lattice mismatches or dislocations^10^. As a result, the widespread application of self-luminous inorganic materials in displays faces significant obstacles.

**Fig. 1:**
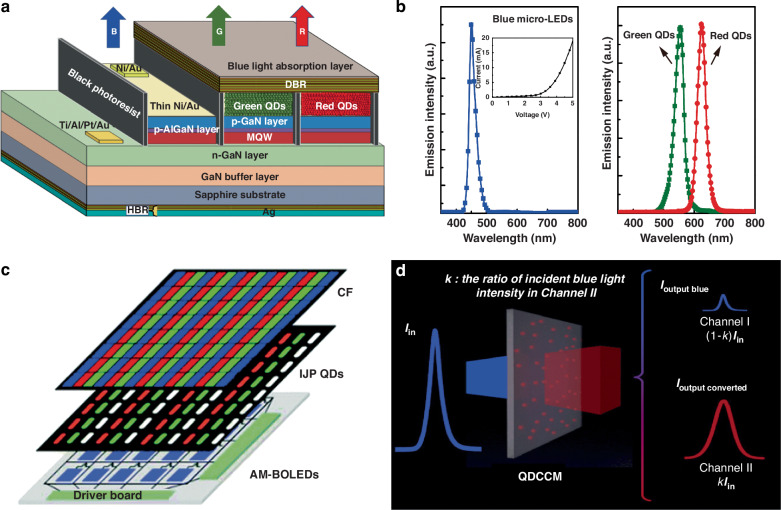
Schematic Structures and Spectral Features of Color Conversion. **a** Schematic configuration of the monolithic full color Micro-LED and **b** the EL spectra of the GaN-based blue micro-LEDs, the emission spectrum of the green and red QDs^14^. **c** A schematic illustration of the architecture for the color conversion OLED display panel^15^. **d** Schematic definition of optical channels for QD CCM^16^

**Fig. 2:**
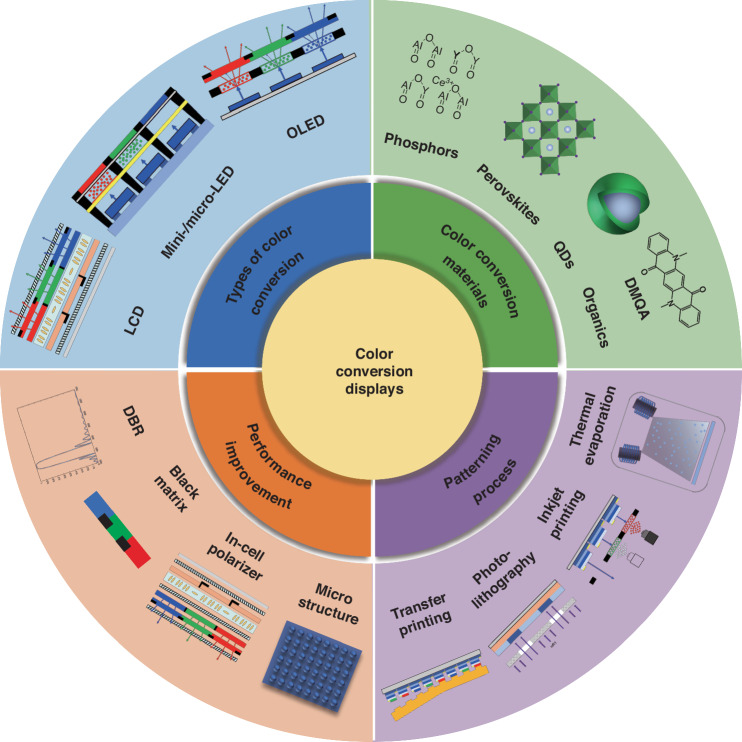
The main content of this review

Color-conversion display technology offers an alternative approach to color reproduction by utilizing high-energy blue light to generate red and green light, enabling full-color representation^11,12^. This technology relies on the photoluminescence properties of light-emitting materials, which can be categorized into incomplete color conversion and complete color conversion. Incomplete color conversion refers to passive luminous displays like LCDs, which is commonly utilized to generate a white backlighting unit (BLU) through the blue LED excitation with red and green color-mixing. The BLU, combined with color filters (CFs) within the LCD panel, enables full-color displays. Although this method eliminates the need for separate red, green, and blue pixels in the LED array, it results in relatively low luminous efficiency due to the loss of CFs absorption^13^, which accounts for a significant two-thirds of energy loss in LCDs. Complete color conversion, on the other hand, utilizes active LEDs such as blue OLED or UV/blue Micro-LED as the excitation source, and coupled with a color conversion process to generate green and red emissions to achieve full-color display (Fig. 1a–c)^14,15^. This approach primarily targets active light-emitting displays and represents a significant focus in contemporary mini/Micro-LED display research. Complete color conversion offers complementary advantages and the potential for high-quality full-color display. However, achieving full-color display through color conversion technology involves complex optical behaviors. To gain a deeper understanding, Chen et al. have proposed a theoretical model of the color conversion principle, with a particular focus on QDs color conversion technology^16^. In the theoretical model, the color conversion process is divided into two logical channels: the blue light transmission channel and the color conversion channel. The blue light transmission channel considers only blue light transmission, scattering, and absorption, while the color conversion channel focuses on the color conversion processes (Fig. 1d). Various parameters, such as color conversion efficiency, blue light transmittance, light intensity, and the photoluminescence quantum yield (PLQY) of the color conversion layers, can be simulated to serve as metrics to quantitatively evaluate color conversion performance and efficacy. Strategies involving material design, structural optimization, and process innovation are developed to enhance and refine these crucial parameters, facilitating efficient, stable, and vibrant color conversion. As shown in Fig. 2, this review article aims to provide a comprehensive understanding of color-conversion displays, covering types of color conversion in displays, color conversion materials (CCMs), patterning processes, and strategies for performance enhancement. Additionally, future prospects and pivotal roles of color conversion is also discussed to guide future research and industry efforts in the development of ultra-high-definition displays.

## Types of color conversion in displays

### Passive luminous display

Color conversion technology is utilized in traditional LCD devices through two main units: the BLU and CFs. In a traditional LCD, the BLU is responsible for illuminating the entire display area. The color of the backlight is determined by a white light source, which has limitations in producing a wide range of color gamut. In a color conversion BLU, a portion of the blue light is absorbed by color conversion materials (CCMs) and then converted into red and green emission. This mixed light, along with the unconverted blue light, serves as a white backlight source^17^.

**Fig. 3:**
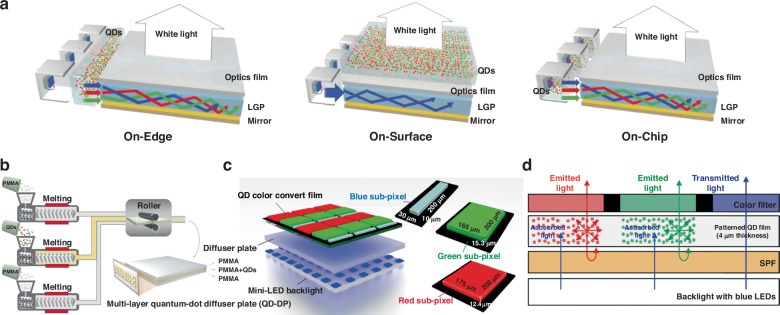
Passive luminous color conversion display structures. **a** Three typical color conversion BLU structures of LCD displays^18^. **b** Multi-layer co-extrusion method for ultra-large QD-DP fabrication^34^. **c** Pixelized QD CCM with different RGB pixel areas illuminated by blue mini-LED backlight^35^. **d** Schematic illustration of the operating mechanism of the patterned-CCM CF-LCD^37^

Figure 3a illustrates three types of color conversion BLU structure: On-Edge, On-Surface, and On-Chip^18^. Color conversion in the BLU expands the color gamut, resulting in a more vibrant visual experience. The first QDs TV, for example, used the On-Edge BLU structure^19^. However, the fragile glass tube used to encapsulate the QDs poses a risk of leakage and thus requires a sealed edge design, making it unsuitable for ultra-narrow border or borderless TV designs^20^. Moreover, the On-Edge solution is too complex and expensive to be implemented in industrial production, and ensuring good color uniformity is also challenging, leading to its phasing out. The On-Surface solution, where the QDs are positioned on top of the light-guide plate (LGP), has emerged as a significant advancement in display technology^21^. By avoiding direct contact with the LED chip, this solution offers superior operational stability compared to the On-Edge solution^20^. Currently, displays based on the On-Surface solution dominate the market. Furthermore, QDs film exhibits compatibility with flexible displays, providing additional opportunities for optimization in terms of stability and the incorporation of materials with integrated scattering functionality. The On-Chip solution, similar to traditional white LEDs where the phosphor is placed on top of the LED chip^22^, involves encapsulating the CCMs onto the surface of LED chips^23,24^. This implementation can partially^22^ or fully^23^ replace traditional phosphors. The On-Chip structure minimizes material consumption, leading to cost reduction and making it highly suitable for integration. However, the On-Chip structure requires high-quality CCMs to ensure stable operation under intense blue light irradiation and high-temperature conditions^18^. If stability issues related to materials and packaging can be addressed, this structure is likely to become a trend in future industrial development. In terms of material optimization, current studies have yielded QD CCMs with high-temperature fluorescence characteristics that meet application standards. Improvements in the fluorescence lifetime of QD CCMs in On-Chip structures can be achieved through methods such as elemental doping^25^, shell coating^26–28^, surface passivation^29,30^, encapsulation using porous membrane substrates^31^, and the formation of polymer composite materials^32^.

To maintain competitiveness and market dominance, the integration of mini-LED BLUs into LCDs has been widely adopted, leading to improvements in display performance such as contrast ratio, peak brightness, bit depth, color space, and power efficiency. In terms of color conversion, a blue mini-LED chip is used as the direct backlight source for the CCM layer, enabling the generation of a white backlight^33^. The device design typically involves integrating CCM layers and a diffuser plate (DP) into a unified component to create an ultra-large QD color conversion diffuser plate (QD-DP). This sandwich-like structure has proven effective in the development of a prototype for a 75 inch LCD TV with mini-LED BLU, exhibiting comparable performance to conventional QD-film-based TVs but with a streamlined structure and reduced cost (Fig. 3b)^34^. The implementation of tri-color pixels with an asymmetric area and precise control of the aperture ratio between QD subpixels has resulted in a wide color gamut of 115.09% NTSC (Fig. 3c)^35^.

In conventional LCDs, more than two-thirds of the incident backlight energy is absorbed by CFs. To improve efficiency and expand the color gamut, a strategy involves shifting the color conversion concept from the BLUs to the CFs within the LCD panel^36^. Color conversion technology can help reduce the light absorption by CFs or even replace the CF layer, thereby enhancing both the color gamut and optical efficiency. Within the CFs of LCDs, a patterned CCM film can serve as a color conversion layer in a specially designed LCD system. Additionally, patterned CCM films placed beneath the CF layer can act as color-converting components and also function as short-pass filter (SPF) elements for optical recycling. The CCM film preserves the narrow bandwidth of the green and red colors before passing through the CFs (Fig. 3d)^37^. It is important to note that the omnidirectional light scattering caused by the CCM introduces complexity when integrating patterned CFs into LCDs, as it interferes with the required polarization state^38,39^. To address this issue, the use of specialized polarization films, such as in-cell polarizers, becomes essential.

### Active luminous display

**Fig. 4:**
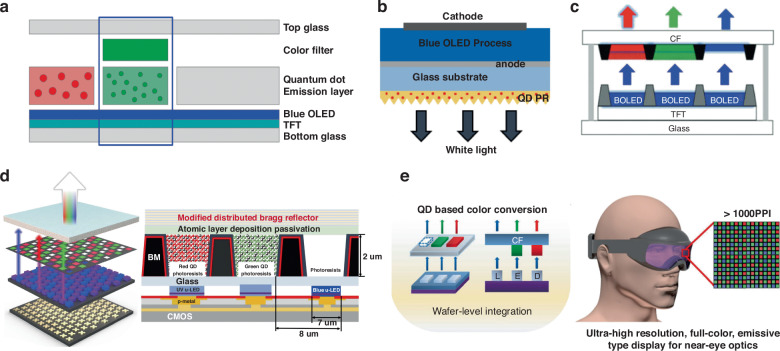
Active luminous color conversion display structures. **a** High green purity QD-OLED with green QD layer and green filter layer^40^. **b** White OLED based on QD photoresist^41^. **c** Inkjet-printed uniform QDs as color conversion layers for full-color OLED displays^15^. **d** Full-color display with blue–UV Micro-LED^46^. **e** High-resolution color conversion Micro-LEDs for near-eye displays^47^

In OLED displays, a simplified panel design can be achieved by implementing only blue pixels, overcoming challenges in large-scale manufacturing. By combining blue pixels with CCMs, full-color OLED displays can be realized. In 2022, QDs made their way into OLED displays, with Sony and Samsung launching QD-OLED technology. QD-OLED displays exhibit a wide color gamut of up to 90% Rec.2020, representing 1.5 times increase in color space compared to traditional OLED displays. However, there is a few of reports of the QD-OLED technology in the research community. In a study by Jeong et al., a green QD CCM layer was integrated onto the indium tin oxide (ITO) layer of a blue OLED, along with a green filter layer to minimize blue light leakage. This structure design can help to achieve high green purity in the QD-OLED configuration (Fig. 4a)^40^. Another approach, as demonstrated by Patel et al., involved the use of a nanoimprint mold consisting of a sapphire master mold and polydimethylsiloxane (PDMS). This mold was used to apply red and green QD CCMs onto a blue OLED (Fig. 4b)^41^. Hu et al. utilized inkjet printing to uniformly print red and green QD CCMs on a CF, resulting in a 6.6-inch full-color QD-OLED display (Fig. 4c)^15^.

The commercialization of Micro-LED display technology is currently being pursued by major companies. However, the manufacturing process for directly emissive Micro-LEDs, whether utilizing AlInGaN or AlInGaP materials, is notably complex. Challenges arise in epitaxial growth, wafer fabrication, mass transfer, and color deviation^42,43^. Moreover, significant discrepancies persist in the luminous efficiency and stability among red, green, and blue Micro-LED chips, alongside formidable obstacles in achieving mass transfer. A promising solution involves the use of blue or UV Micro-LED chips with CCMs to achieve a precise manufacturing process, improve the production takt, and address the challenges of drive circuit design and color stability^44^. When a blue Micro-LED is used as the emitter, it is coupled with red and green CCMs to achieve a full-color display. When an ultraviolet (UV) Micro-LED is used as an emitter, the visible primary colors are achieved by color conversion through red, green, and blue CCMs. The main advantage of the UV Micro-LED approach is the higher light absorption by the CCMs at the UV wavelength, and better color gamut that can be achieved without the blue leakage issue. The overall energy efficiency of the UV Micro-LED approach can be improved significantly, leading to a longer battery life, especially for augmented and virtual reality (AR/VR) applications with limited energy capacity in compact systems^45^. However, there is a possible issue arising from the UV approach as the UV light is known to affect sleep by increasing eye fatigue and suppressing melatonin secretion. Precision pattern-making techniques like inkjet printing and photolithography can be extended to color conversion Micro-LED display devices. Lee et al. investigated the photolithographic process to fabricate the CCM layer^46^. Under mixed excitation of blue LEDs and UV Micro-LEDs, they achieved excellent uniformity in red and green subpixels and enhanced color efficiency (Fig. 4d). Junho Bae et al. achieved a full-color Micro-LED array with 20 μm RGB spacing by bonding red and green QDs as color conversion layers atop blue GaN Micro-LEDs (Fig. 4e), expanding QD-based color conversion Micro-LEDs into the realm of high-resolution near-eye displays. This was facilitated by high-resolution color conversion Micro-LEDs, utilizing solvent-free CCM patterning at lithographic levels with an elastic PMMA mask. Their method represents a promising solution for near-eye displays incorporating QD, gallium nitride, and silicon electronic components in innovative applications^47^. At present, major companies like Innolux, Sitan, Roysolve, and Saphlux are adopting the color conversion approach to commercialize their Micro-LED displays.

## Color conversion materials

CCM plays crucial roles in the performance of color-conversion displays. Currently, available CCM options include organic fluorophores, inorganic phosphors, QDs, and metal halide perovskites (MHPs). Among them, phosphors have been widely used as CCMs in phosphor-converted LED lighting and display technologies for over 70 years^17^. Phosphors are inorganic crystals host materials doped with rare-earth or transition metal activator ions. Rare-earth or transition metal activator ions have been partially substituted within the host structure and act as luminescence centers^48^. They are commonly coated on or near the surface of the blue LED chips to produce necessary spectrum for white light generation and set the minimum bar for the color-rendering index, correlated color temperature and efficacy^49^. Phosphors has advantages in quantum yield and thermal/chemical stability. However, the relatively large particle size of several micrometers (generally 5–10 *μm*)^48^, the size-dependent luminance homogeneity^50^, and the large full width at half maximum (FWHM) hinder their application in ultra-high-definition full-color display^51^.

Organic fluorophores are another potential option as CCMs. They are typically organized in a host-guest system, where the host material strongly absorbs blue LED wavelengths and transfers energy to green and red dopants with narrow photoluminescence emission bands. One advantage of organic fluorophores over inorganic materials is their compatibility with vacuum evaporation processes, which are well-suited for manufacturing high-resolution displays using high pixel shadow mask^52^. However, the dopant concentration in organic fluorophores is limited to a low level, resulting in poor color conversion efficiency.

Currently, QDs (recipient of the 2023 Nobel Prize in Chemistry), are replacing phosphors as the preferred color conversion media to enhance the color performance of various displays, including LCDs, OLEDs, and Micro-LEDs. QDs are synthesized through chemical reactions and typically have a nanometer-scale size. These quantum-confined nanocrystals offer the potential to take current display technologies to the next level by increasing brightness, expanding the color gamut, and improving contrast ratio. The use of QDs in displays dates back to 2013 when Sony launched the first commercial 65” Cd-based QD-LCD display. In 2022, QDs were successfully incorporated into OLED displays by Sony and Samsung. Nowadays, Innolux, Sitan, Roysolve, and Saphlux are all using QDs to make the color conversion Micro-LED displays. Color conversion with QDs has maintained a competitive advantage over LCD and OLED displays in terms of performance for some time, and we have also witnessed a significant decline in pricing and assembly costs for QD-enhanced display systems. There is great hope for the usage of QDs in Micro-LEDs, as they hold tremendous potential for this emerging display technology. In recent years, MHPs have emerged as a promising candidate that can fully satisfy the color gamut requirements of Rec.2020 for ultra-high-definition displays^53^. In addition, MHPs can be synthesized with a simple and low-cost way, they have been found lots of applications ranging from photovoltaics, LEDs, and lasering^54–58^. However, the stability and scalable fabrication are still issues for the industry.

### Optical properties

**Fig. 5:**
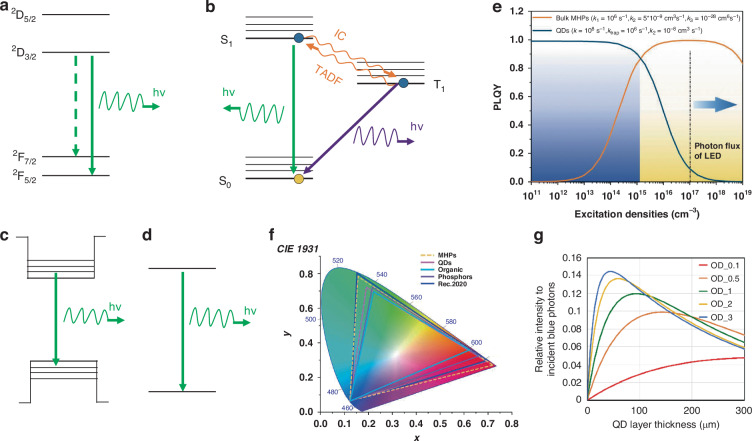
Mechanism and performance of CCMs. Emission mechanisms of (**a**) phosphors. **b** organic fluorophores. **c** QDs. **d** Bulk MHPs. **e** Excitation-density-dependent PLQY plots of bulk MHPs and quantum-confined QDs. **f** The CIE chromaticity diagram of color-conversion displays made of different CCMs. **g** The emitted photons versus QD CCM layer thickness under different optical density values^81^

It is crucial that CCM suitable for display applications must possesses adaptive optical properties, including high PLQY, narrow FWHM, and strong absorption of blue light. These optical properties are primarily determined by the excitation/emission processes, which vary among different types of CCMs as organic fluorophores, inorganic phosphors, QDs, and MHPs. The optical properties of phosphors, for instance, arise from the interactions between a crystalline host material and an activator ion, typically a rare-earth or transition metal ion. The activator ion is partially substituted within the host structure and acts as a luminescent center. The electronic transition occurs through parity-forbidden transitions involving d- or f-orbitals of the dopant ions upon excitation. The electronic transition occurs through parity-forbidden transitions involving d- or f-orbitals of the dopant ions upon excitation (Fig. 5a)^59^. This transition process is with a relatively long decay life time of microseconds to milliseconds, known as phosphorescence. In organic fluorophores, the excitation/emission process involves transition between the singlet ground state (S_0_) and singlet (S_1_), or triplet (T_1_) excited states together with charge transfer (CT) interactions, known as the fluorescence, phosphorescent, or thermally activated delayed fluorescence (TADF) (Fig. 5b). The vibronic coupling in fluorescent and phosphorescent organic emitters often induces quite broadband and asymmetric photoluminescence spectra^60^. In QDs or MHPs semiconductors, transition is through a radiative band-to-band luminescence process (bulk) or an excitonic process (QDs), as shown in Fig. 5c, d. The emission properties are mainly dependent on the crystal structure of bulk semiconductor and the internment of electron hole pairs (excitons) within the nanocrystal grain boundaries^61^. For example, in bulk MHPs, band-to-band transition is determined by the conduction band minimum (CBM) and valence band maximum (VBM). CBM is formed by the unoccupied Pb 6*p*-halide n*s* antibonding orbitals and is mostly affected by the Pb *p* orbital, whereas the VBM is composed of the filled Pb 6*s*- halide n*p* antibonding orbitals^62^.

One of the most important optical properties of a CCM is the ability to efficiently absorb and down-convert photons to longer wavelengths. The efficiency of this process is described by the charge-carrier recombination process, as given by Eq. (1).

1 $$\frac{{dn}}{{dt}}=-{k}_{1}n-{k}_{2}{n}^{2}-{k}_{3}{n}^{3}$$

where ${k}_{1}$, ${k}_{2}$ and ${k}_{3}$ are the first-, second-, and third-order recombination rate constants associated with defect-assisted (monomolecular), radiative (bimolecular) and Auger (three-body) recombination processes, respectively, and *n* is the photogenerated carrier density. The PLQY of the bulk semiconductor is then given by the Eq. (2).

2 $${PLQY}=\frac{{k}_{2}n}{{k}_{1}n+{k}_{2}{n}^{2}+{k}_{3}{n}^{3}}$$

In QDs with crystal sizes smaller than exciton Bohr diameters, the first-order radiative excitonic recombination competes with first-order non-radiative recombination through charge- or exciton-trapping, and with second-order non-radiative Auger recombination of exciton-electron/hole or exciton–exciton annihilation^63^. The recombination kinetics and PLQY can be described by Eqs. (3) and (4), respectively^64^.

3 $$\frac{{dn}}{{dt}}=-{k}_{{trap}}n-{kn}-{k}_{2}{n}^{2}$$

4 $${PLQY}=\frac{k}{{k}_{{trap}}+k+{k}_{2}n}$$

where ${k}_{{trap}}$, $k$, and ${k}_{2}$ are recombination rate constants of non-radiative monomolecular recombination, radiative monomolecular recombination, and Auger recombination, respectively. According to Eq. (2), PLQY is excitation density dependence, and mainly affected by the ratio of radiative recombination rate over non-radiative recombination rate.

Figure 5e illustrates the PLQY as a function of excitation density for typical bulk (MHPs) and quantum-confined emitters (QDs). In bulk semiconductor like MHPs, PLQY close to unity can only achieved at specific excitation density. Typically, for example, this occurs at a photon flux range of 10^15^ ~ 10^19 ^ cm^−3^. At this excitation densities, radiative bimolecular recombination dominates the process due to the filling of charge traps as the carrier population increases. However, beyond a certain threshold, nonradiative Auger recombination becomes more prominent, leading to a decrease in PLQY. In contrast, in QDs, Auger processes are negligible at low excitation densities. The PLQY in QDs is given by $k/({k}_{{trap}}+k)$, where $k$ represents the radiative rate and ${k}_{{trap}}$ represents the nonradiative trapping rate. Importantly, this expression is independent of the excitation density. Thus, by engineering trap defects, high PLQYs can be achieved at low excitation intensities (<10^16^ cm^−3^). Indeed, near-unity PLQYs have been demonstrated in a range of QDs and perovskite nanocrystals^65,66^. However, under high excitation densities, Auger recombination becomes highly efficient in QDs due to the relaxation of momentum conservation requirements and increased Coulomb interactions^67^.When the excitation density exceeds a threshold, Auger recombination dominates the carrier dynamic process of QDs, leading to a dramatic decrease of the PLQY. The excitation density dependent PLQY must be taken into consideration in real application in displays. Most display implementations can be designed with blue fluence of 0.1 ~ 1 *W.cm*^−2^, corresponding to an excitation density of 10^17^ ~ 10^18^ cm^−3^^ 68^. While in high brightness high-resolution micro-displays, high photon flux (~1–10 *W.cm*^−2^) is needed. The corresponding excitation intensity is ten times high (10^18^ ~ 10^19^ *cm*^*-3*^). The flux exceeds the threshold value of excitation density for Auger recombination in QDs. Therefore, it is a challenge to design QDs with suppressed second-order non-radiative recombination for color-conversion displays. It is a challenge to design QDs with suppressed second-order non-radiative recombination for color-conversion displays. Previous studies have directed effective ways to reduce the Auger recombination by changing the size or shape of the core and shell, introducing an alloy interfacial layer between the core and shell, and controlling of the geometry-dependent dielectric screening^67,69^.

In color-conversion display applications, color purity holds equal importance alongside the PLQY as it directly influences the color gamut achievable by the display. The characterization of color purity is commonly done through the FWHM of the luminescent materials’ emission spectrum. A narrow FWHM and appropriate emission wavelength are crucial in determining the coverage of the Rec.2020 color space. In the case of phosphors, the FWHM is primarily governed by the strength of electron-phonon coupling and the equilibrium distance between the potential energy surfaces of the ground and excited states. More specifically, it is strongly influenced by the ligand field effect combined with lattice vibrations^49^. These factors play a significant role in shaping the spectral characteristics and color purity of the phosphor materials used in color-conversion displays. The U.S. Department of Energy (DOE) recommends that phosphors used in displays have a FWHM of 30 *nm* to maximize energy efficiency. However, achieving such a narrow FWHM of 30 *nm* is challenging for inorganic phosphors. This is because narrower FWHMs would require each dopant to exist in the exact same chemical environment, which is difficult to achieve^70^. In the case of QDs, their lattice provides a wide range of vibrational modes known as lattice phonons. These phonons span from low-energy acoustic phonons to moderate-energy optical phonons^71^. Compared to organic molecules, the phonon energy in QDs is much smaller. Additionally, the numerous chemical bonds within a typical core/shell QD are approximately equal, resulting in the input energy from an electron-hole pair being distributed throughout the entire QD without significantly affecting its bonding^18^. As a result, typical QDs exhibit a comparatively narrow and symmetric emission peak at room temperature, rather than having a low-energy tail. However, the emission peak width of QDs is influenced by factors such as size, crystal structure, morphology, surface structure (including ligands), and epitaxy of shells^72^. In practical application, the uniform size distribution and weak electron-phonon coupling of QDs enable them to emit highly pure colors with narrow emission bandwidths ranging from 25 to 40 *nm*^18^. In the case of MHPs, the linewidth of the PL spectrum is mainly affected by the interactions between charge carriers and longitudinal optical phonons via Fröhlich interactions, rather than impurities or defects^73^. Therefore, the FWHM and emission color of MHPs are not strongly dependent on their size but rather on their crystal structures and compositions, resulting in a low FWHM of around 20–30 *nm*^53^. For comparison, organic emitters generally have FWHMs >40 *nm*^74^. Figure 5f illustrates the color gamut of displays using phosphors, organic emitters, Cd-based QDs, and MHPs as red and green color conversion materials (CCMs). Organic emitters with their large FWHMs (>40 *nm*) cover only 65% of the Rec.2020 color space. QDs, with FWHMs between 20 *nm* and 30 *nm*, achieve around 8–90% Rec.2020 coverage due to non-ideal size and distribution control. In contrast, MHPs can cover ~100% of the Rec.2020 color space.

The third requirement for color-conversion displays pertains to light absorption, particularly at the wavelength of blue LEDs. The extent of absorption plays a crucial role in determining the thickness of the CCM layer, which, in turn, affects both the color conversion efficiency and the color gamut of the display. Ideally, the CCM should exhibit a strong absorption in the range of 450–460 *nm* to minimize blue leakage and maximize the desired efficiency of red or green color conversion. In organic, the build of a large in-plane π-π* transition dipole moment would lead to a large molar extinction coefficients^52^. The recently reported thermal evaporated perylene derivative bis(2,6-dimethylphenyl)perylene (DMP) exhibits an exceptional absorption coefficient of 1.9 × 10^5^ cm^−1^ at 458 *nm*, which corresponds to an optical density of 3.8 for a 500 *nm* film^52^. Although both Cd-based QDs and MHPs has broadband absorption and absorption coefficient of 10^5^cm^–1^, their application in the color-conversion display generally requires 10 *μm* or more for an optical density exceeding 2^75^. This is because that QDs and MHPs are often need to be diluted in a polymer matrix, the extinction coefficient is proportional to the volume of the QD in matrix^76^. The optical properties of CCMs can be significantly improved through the introduction of microstructures or micropatterns. Particularly in the color conversion of QDs, where microstructures or micropatterns can reduce the required QDs concentration for the same photoluminescence intensity, minimizing light reabsorption events^77–79^ In some cases, if the CCM has a limited Stokes shift, self-absorption is a critical factor affecting the efficiency of color converters^80^. Therefore, it is necessary to evaluate the optimized thickness. A small fraction of the emitted light can therefore get re-absorbed by the CCM itself, which in turn gets converted and emitted. The probability for re-absorption is influenced by factors such as the spectral overlap, the QD solid loading, and the optical outcoupling efficiency. Figure 5g shows the optimum CCM thickness under different optical density values, which is evaluated according to Eq. (5)^81^.

5 $${I}_{{red}/{green}}={\int }_{0}^{d}{I}_{0}\frac{{\nu }_{{red}/{green}}}{{\nu }_{{blue}}}A \% \,\cdot \,{\alpha }_{{blue}}{e}^{-{\alpha }_{{blue}}x}({e}^{-{\alpha }_{{red}/{green}}(d-x)}){dx}$$

where ${I}_{{red}/{green}}$ is the intensity of red/green photons, *d* is the CCM layer thickness, ${I}_{0}$ is the incident intensity of blue photons, $A \%$ is the percentage of absorbed blue photons and re-emit red/green photons, ${\alpha }_{{blue}}$ is the blue light absorption coefficient of CCM, *x* is the position, ${\nu }_{{red}/{green}}$ and ${\nu }_{{blue}}$ are the frequencies of red/green and blue photons, respectively. As the blue absorption in CCM layer gets higher, the peak intensity of the emitted red or green photons will get higher and the optimal thickness will become thinner as well. Thus, it is not always beneficial to increase the thickness of color conversion layer in order to increase the CCM emission. It is important to find out their best thickness to balance the absorption and re-emission of the specific color of photons^81^. In order to enhance the light absorption to prevent the blue leakage, additional approaches have also been used. The first one is the use of band selective reflectors such as distributed Bragg reflector (DBR), hybrid Bragg reflector, and stacked cholesteric liquid crystal^14,82^. The second is to add scattering particles that can increase the effective optical path length and therefore absorption^83^.

### Reliability

As a color conversion medium for color-conversion display applications, the color conversion efficiency, color purity, and light absorption of CCMs are prone to deteriorate during the operation. The temperature of the QD CCM can go up to 110 °C when irradiated with an intense blue light flux of *20* *W.cm*^*-2*^, leading to thermal quenching, linewidth broadening, and even failure^68^. In terms of the linewidth broadening, red and green QDs have been reported to broaden 1 nm per 15 °C because of exciton scattering with acoustic and LO phonons, while phosphors broaden 1 *nm* per 10 ~ 20 °C in the temperature range of 25 ~ 200 °C^18,84^. Spectral broadening absolutely affects the color gamut of the color-conversion display. For instance, when the FWHMs of the green (528 *nm*) and red (637 *nm*) CCMs increase from 20 *nm* to 45 *nm*, the color gamut decreases from 95.7% to 87.2% of Rec.2020 accordingly^85^. In practice, this translates into very challenging operating conditions for QDs where still today dedicated development is required to reach satisfactory long-term performance.

In order to alleviate the reliability issues, suitable panel designs are taken into account as it has a key influence on the required blue power density and many high-brightness displays can be made below 1 *W.cm*^*-2*^. For example, in the design of BLUs in LCD display, the QD CCM is suggested to be placed remotely from the blue LED to allow the QDs to operate with blue light intensities at or below the threshold. Therefore, the On-Surface configuration is preferred in comparison with the On-Chip arrangement in practical^18^. This threshold blue light flux of 1 *W.cm*^*-2*^ is suitable for large displays, TVs, smartwatches, automotive and most other direct-view displays. The main application that falls out of this range is micro-display, where the required millions of nits translate into ~5–10 *W.cm*^*-2*^ blue light flux^86^. Cd-based QDs have historically shown good results in terms of photostability under high blue light flux, and they are quite widely adopted in color-conversion display prototypes. However, MHPs are reported with poor light stability due to light-induced ion migration and phase segregation, especially in the case of mixed-halide perovskite^30,87–89^.

A common way to protect the CCMs from moisture and oxygen relies on the packaging. CCMs are need to package in a solid-state matrix composed of materials such as polymers, ionic crystals, or inorganic particles. In the case of QDs, when mixing or embedding them into a matrix, it is prone to induce detachment of surface ligands, leading to uneven dispersion and aggregation and thus the luminescence quenching and degradation of the optical properties. The straightforward approach is using the surface ligand engineering, which focuses on the improvement of the affinity between CCMs and the outer matrix or employing cross-linkable ligands to directly form the coating. With proper choose and design of the surface ligands, the as-obtained CCMs can exhibited excellent acid resistance, base resistance, photostability, and thermostability^90^. Mechanical dispersion and in-situ polymerization are two methods widely used to package CCMs into a matrix film^91^. The principle of the mechanical dispersion is to directly mix the CCMs dispersion into a polymer solution by a mechanical process, such as magnetic stirring, or sonication. The in-situ polymerization is to blend CCMs dispersion with the monomer solution, followed by a polymerization process of the monomer to solidify the polymer matrix. A simple, low-cost, highly repeatable, and green package process is the direction of future research to promote CCM s for large-scale commercial applications.

According to the Restriction of Hazardous Substances (RoHS), the directive limits the concentration of hazardous substances to ≤100 *ppm* for Cd and ≤1000 *ppm* for Pb by weight. Cd QDs are more strictly regulated than MHPs, and organic emitters and Cd- or Pb-free QDs are not restricted by RoHS. The disposal problem of these QDs and MHPs becomes especially intractable when the device’s lifetime is reached. The comparison of the different CCMs is given in Table 1.

**Table 1:** Comparison of the characteristics of different CCMs for color-conversion displays

CCMs	Phosphors	Organic	QDs	MHPs
Blue absorption	poor	excellent	fair	good
Thickness required	~100 *μm*	~0.5 *μm*	~10 *μm*	~5 *μm*
Photostability	excellent	fair	good	fair-good
Thermal stability	good	poor	fair	fair
Quantum yield	good	fair	good	good
Color purity (FWHM)	poor (100 *nm*)	fair (40 *nm*)	good (30 *nm*)	excellent (20 *nm*)
Toxicity	-	-	Cd (≤100 *ppm*)	Pb (≤1000 *ppm*)

## Patterning process of color conversion material layer

In full-color displays, the sub-pixel can be varied from 50 ~ 100 *μm* for large-panel displays to 3 ~ 5 *μm* for micro-displays. Techniques should be developed to pattern the R/G/B subpixels in an efficient and cost way. At present, there are four typical patterning technologies. They are thermal evaporation, inkjet printing (IJP)/Electrohydrodynamic printing (EHD), photolithography, and transfer printing.

### Thermal evaporation

**Fig. 6:**
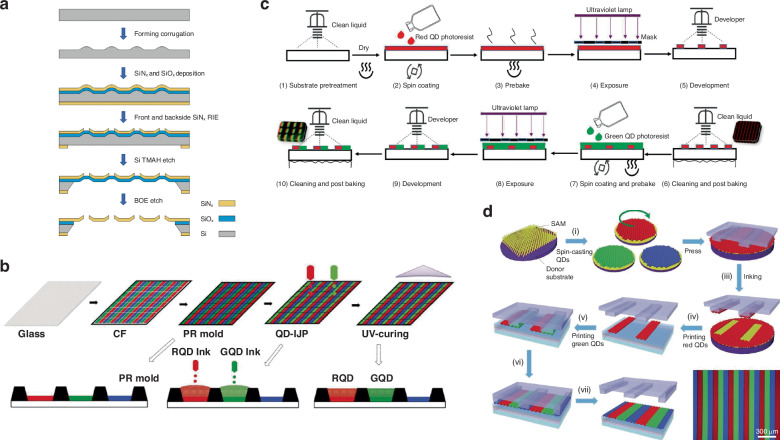
Schematic illustration of different patterning processes. **a** Fabrication process flow of corrugated SiNMx mask for thermal evaporation^130^. Schematic illustration of typical patterning process **b** Inkjet printing of QDs for OLED^15^ (**c**) Photolithography process of red and green QDs^101^ **d** Transfer printing for patterning of QDs^109^

Thermal evaporation is a well-known thin film deposition technique in semiconductor and display industry. CCM layer can be coated from the source material evaporating in a high vacuum chamber with high temperature heating. This method is especially applicable for material with low melting points. The maturity and reliability of thermal evaporation make it a competitive route for large-area and scale-up industry fabrication. During the thermal evaporation process, shadow mask is needed to realize the pixelation. The fine metal mask (FMM) currently used to pattern RGB side-by-side OLED displays for smartphones is limited to resolutions under 1000 PPI by the finite thickness of the metal foil (typically over 10 *μm*). As an alternative to FMM, silicon nitride masks (SiNMs) were developed for patterning OLED micro-displays^92^. These shadow masks comprise a micron-thin, free-standing silicon nitride (SiNx) membrane stretched on a solid silicon frame. The fabrication process of SiNMs is illustrated in Fig. 6a. Owing to the much-reduced mask thickness compared to FMM, the aperture density achievable by the SiNMs was significantly increased to >3000 PPI for patterning of R/G/B organic CCMs, showing its great potential in high-resolution micro-displays for AR/VR/XR^93^.

### Inkjet printing/ electrohydrodynamic printing

IJP is a drop-on-demand technology that has attracted interests as it is highly desirable of process-controllable, flexible, mask-free, and material-compatible. During the IJP process, CCMs are often dispersed in a polymer matrix or a UV-adhesive to achieve the thickness of interest and encapsulation. The IJP process of QDs CCM for OLED display is illustrated in Fig. 6b^15^. With this process, micrometer-thick QD CCM with a uniform surface morphology based on a QD-Blue OLED architecture was fabricated, and a 6.6-inch full-color display with 95% Rec.2020 color gamut and wide viewing-angles was successfully demonstrated. Using IJP technology and preset templates, CsPbBr_3_ green perovskite and CdSe red QDs were patterned on blue Micro-LED with a single pixel size of 200 *μm* x 100 *μm*. A prototype of a full-color-converted Micro-LED display with a color gamut up to that of 129% NTSC was reported^94^. However, the mixture of the CCM with polymer matrix is prone to aggregation and nonuniform dispersion. Careful control of the complex hydro-and thermodynamic processes is also needed to suppress the coffee ring effect.

IJP has drawbacks of its accuracy and limitation, which is strongly dependent on the performance of the nozzle and the properties of the ink. The sub-pixel size from IJP is around 10 ~ 30 *μm*. Electrohydrodynamic (EHD) makes use of the electrostatic field between the nozzle and substrate to regulate the nanoparticle-contained stream and exceed the regular capillary force^95^. It is suitable for high-resolution and high-precious deposition, with resolution down to the sub-micrometer level^96–100^. The high-resolution of EHD down to 1 *μm* making it suitable for high-definition AR/VR/XR micro-display applications. The drawbacks of the EHD technique are that it requires a conductive substrate to generate the necessary electric field, which limits the applicability of this method. Moreover, applying an electric field to drive the formation of the material also causes a side effect on the ink itself, especially for materials with electrically conductive.

### Photolithography

Photolithography is an industry method suitable for wafer-level manufacturing. Photolithography method has been widely studied and applied to pattern organic, QDs, and MHPs for color-conversion display. During the preparation of QD color conversion film, the photolithography process is to accurately transfer the pattern of the lithographic mask onto the surface of the polymethylmethacrylate (PMMA) substrate through a series of processing processes such as spin coating QD photoresist, exposure, and development, as shown in Fig. 6c^101^. Using the photolithographic and etching technique, a full-color Micro-LED with 1588 PPI was obtained using organic CCMs^102^. However, CCM solution should be delicately managed as the incompatibility between CCMs and photoresists leads to the degradation of CCM optical property^103^. Practically, ligand-exchange treatment is useful for QD CCMs to change the surface chemical state to adapt the process^104^. Attention should also be paid to the strong UV light absorption of CCL when exposure to UV light during the photolithography process, which greatly affects the resolution of the pattern. This situation will become worse when the aspect ratio (thickness to the side length) of the QDPR pattern increases. Multiple exposure has been demonstrated to be feasible to address these issues^91^.

Beyond the traditional photolithography process, direct laser writing, mask-free projection lithography, electron-beam lithography and X-ray lithography have been introduced for the pixelation of CCMs^103,105–107^. Among them, laser direct writing method is promising for ultrafine feature size control. By manipulating the power and spot size of the laser, a nice balance between pattering resolution and processing speed can potentially be achieved for mass production, and it exhibits high flexibility as a mask-free process with lower equipment requirements because it utilized a UV laser as the excitation source^108^.

### Transfer printing

Transfer printing assembles the CCM on a prepatterned mask surface or into a spatially organized shape and then transfers the patterned layer of the material to the target substrate. It is mainly used for QD CCM pixelation. During the transfer printing process, the difference in affinity of the master and target substrate for QD materials is the key to this process. Among transfer printing method, nanoimprinting technology is a popular micro-nano processing technology, the resolution can be up to tens of nanometers.

The transfer printing is shown in Fig. 6d. With this solvent-free transfer printing process, a full-color, large-scale QDs for displays in a relief printing form was demonstrated^109^. An intaglio transfer printing was also reported to fabricate RGB QD pixels with a resolution of up to 2460 pixels per inch^110^. The main advantage of the transfer printing is its solvent-free process. However, it requires a mask for specific patterns, which reduces the flexibility of the process and diminishes the forming accuracy after repeated use of the mask, limiting its application prospects. Although higher resolution has been achieved, few studies have been done to realize RGB full-color-conversion displays. Because the thickness of CCM films prepared by transfer printing method is mostly in the range of hundreds of nanometers, it can lead to significant light leakage within the CCM layer, thereby constraining its advancement^111^.

Table 2 compares different patterning processes for color-conversion displays. In general, IJP is compatibility with solution processed CCMs that are cost-efficient for mass production in the display industry. The CCM ink of IJP needs to be well designed to prevent the coffee ring effect and the block of the nozzle. The pixel size is normally limited to tens of micrometer level (>10 *μm*), which is not sufficient for AR/VR/XR applications. EHD can significantly reduce the pixel size to even sub-micrometer (>1 *μm*), but it has drawbacks of the material selection, and the production efficiency and capacity for large-scale fabrication has not been proven yet. Photolithography is technically mature, easier to operate, and easy to industrialize and commercialize on a large scale, but CCMs may be damaged by the solvent used in the photolithography process, deteriorating the optical performance of displays. Transfer printing methods are also feasible for high-resolution fabrication of CCMs. However, it is relatively rare in practical applications, mostly due to its low thickness, contact contamination, difficult alignment, and peeling of the nanoimprint primer.

**Table 2:** Comparison of different patterning processes for color-conversion displays

Patterning process	Thermal Evaporation	IJP/EHD	Photolithography	Transfer Printing
Materials	Organic, MHPs	QDs, MHPs	Organic, QDs, MHPs	Organic, QDs, MHPs
Materials friendly	good	good	poor	poor
Feature size	several *μm*	several *μm*	several *μm*	hundreds *nm*
Mask	yes	no	yes	yes
Thickness	~*μm*	~*μm*	~*μm*	~*nm*
Cost	high	low	high	low
Programmable	no	yes	yes	no

## Performance improvement

### Optical microstructures

**Fig. 7:**
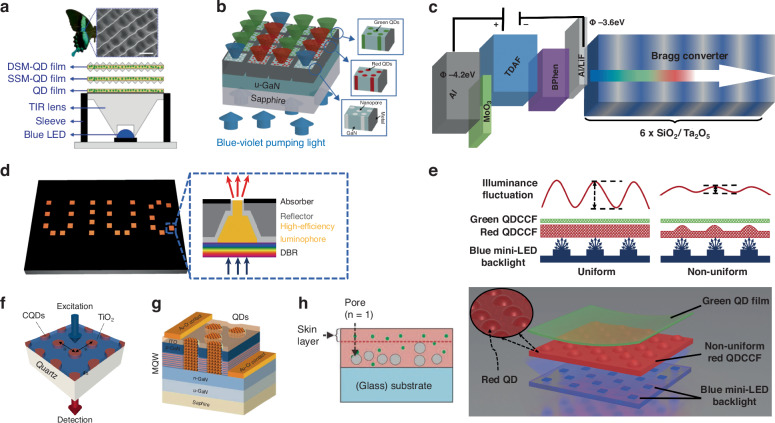
Optical microstructures for performance improvement. **a** Papilio blumei butterfly and the micro concave cone array of its wings (Up); Schematic of LED devices with QD, SSM-QD, and DSM-QD films (Down)^117^. **b** Schematic diagram of RGB array structure with red and green QDs selectively loaded into a blue-violet GaN array^118^. **c** Schematic of the Bragg White-OLED concept^119^. **d** Micropixels with luminescent concentrator design^119^. **e** QD color conversion film with nonuniform thickness^121^. **f** Schematic of the 2D photonic crystal structure^123^. **g** Schematic representation of a photonic quasi-crystal LED hybridized with QD color converters^114^. **h** Schematic overview of the foamed hybrid color conversion films^113^

The surface structure of the CCM layer is the most commonly used strategy and is also a strategy that runs through the development of displays. Surface structure enhancements encompass the scattering effect of textured surfaces, wavelength-dependent reflection and transmission spectra of functional layers, and plasma effect of metal nanoparticles^91^. Examples include randomly imprinted corrugated patterns or corrugated microcavities^112^, lens arrays^113,114^, and photonic crystal structures^115^, all demonstrating effective enhancement of opto-external coupling. Han et al. devised a high-performance microlens array thin film for down-converted white OLED, utilizing a soft imprinting method with a breathing graphic pattern. White OLEDs incorporating lens array films exhibited significantly improved optical coupling efficiency and high-quality white light, with power efficiency improvements of 1.35 times and 1.86 times, respectively. Additionally, achieving high-quality white light with a color rendering index of 84.3 further demonstrated the effectiveness of microlens array films^116^. Inspired by nature, A micro-concavity array (MCA) composite was introduced, mimicking the surface texture of a Phoenix butterfly’s wing and combining it with a QD composite. This resulted in single-sided micro-structured QD (SSM-QD) and double-sided micro-structured QD (DSM-QD) composites to enhance the color conversion efficiency of the QD-LED. Optimization of MCA diameter, aspect ratio, and spacing led to improved LED device conversion efficiency using SSM-QD and DSM-QD composites, achieving enhancements from 19.98% to 21.59% and 21.78%, respectively. The scattering and reflection effects of the MCA composite were attributed to the improved performance (Fig. 7a)^117^. Kang et al. employed nanoporous GaN to enhance the color down-conversion of micro-LEDs. Nanoholes etched on the GaN material loaded with colloidal QD material in the pore structure demonstrated significant increases in absorbance due to enhanced light scattering. Green and red emission light conversion efficiencies reached 96% and 100%, respectively, under multiple light scattering within the porous structure (Fig. 7b)^118^.

Efficiency and quality enhancements can also be achieved through novel structures, such as the “Bragg converter” proposed by Daskalakis et al.^119^. This converter consists of a thin blue emission layer coupled to the photon Bragg mode of a dielectric distributed Bragg reflector, effectively enhancing photon emission and external coupling at selected wavelengths across the visible spectrum (Fig. 7c)^119^. Braun et al. presented a display pixel utilizing QD CCM embedded in a polymer matrix, combined with a light-emitting condenser design. The pixel design achieved high photon extraction efficiency and environmental contrast, utilizing the narrow-band emission of QDs to achieve photon recycling without requiring a light-absorbing CF. The proposed structure achieved 40.9% extraction efficiency for a single pixel (Fig. 7d)^120^.

Microstructures on the CCM layer not only enhance optical efficiency and color rendering performance but also contribute to improved light output uniformity in backlight display modules and enhanced imaging quality. Chen et al. designed a raised point structure on the color conversion layer, corresponding to the backlit mini-LED light bar, to achieve more uniform light output in the backlight module, improving brightness uniformity (Fig. 7e)^121^. The light efficiency of color conversion can also be significantly improved in the form of photonic crystals^122^. When the light with the edge wavelength of the photon band is incident on the photonic crystal material, the excited photon is absorbed by resonance, thus enhancing the emission intensity. The two-dimensional square lattice photonic crystal fluorescence material is considered to be the most advanced platform at present because it is not only efficient, but also has immunity to excitation polarization. Lee et al. improved the structure and material of photonic crystals respectively. TiO_2_ was used to replace the main chain material of photonic crystals to improve the refractive index contrast. Secondly, surface flattening was achieved by removing excessive colloidal QDs from the surface. Compared to the reference material, the upgraded photonic crystal material exhibits ~59 times the absorption (in the simulation) and ~7 times the enhanced emission (in the experiment) (Fig. 7f)^123^. Krishnan et al. proposes an efficient hybrid 12x photonic quasicrystal multi-quantum-well white LED geometry that substantially improves external coupling efficiency while retaining the good electrical properties of pattern-less devices. This architecture allows electrically injected carriers to be efficiently coupled to the QD via non-radiating resonant energy transport, while allowing near-field coupling between the blue light field and the emitter within the LED. This resulted in record effective quantum yields of 123% for monochromatic quantum dots and 110% for white LEDs (Fig. 7g)^114^.

In addition to making improvements on the surface or outside of the CCM layers, the internal porosity strategy can also improve the efficiency of the CCMs by creating micropores or micropores inside the QDs color conversion composite material as a scattering medium to increase the absorption of excited light and enhance light extraction. Yu and colleagues described a supercritical CO_2_ foaming technique forming microporous structures in PMMA composite films embedded with CdSe/ZnS QDs, significantly improving the overall photoluminescence intensity of QD-based CCMs compared to composites with non-porous structures. The micropores increase the angular diffusion of UV/blue excitation within the polymer matrix, and the optical scattering results from the refractive index contrast between the micropores and the polymer matrix. However, this method maintains a high transmittance of up to 80% in the visible light range. For the porous network, the maximum photoluminescence intensity enhancement factor measured was 6.6 compared with the non-foaming QD/PMMA composite film (Fig. 7h)^113^. Through these external/internal light extraction strategies, the great potential of global energy conservation and sustainable development can be maximized.

### In-cell polarizers

**Fig. 8:**
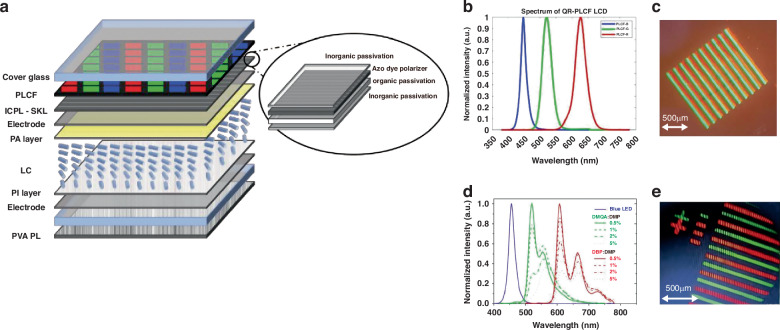
Photoluminescent CF and in-cell polarizer desgin of LCD. **a** The structure of LCD device with a photoluminescent CF and in-cell polarizer^127^. **b**, **c** The photoluminescence spectrum and optical microscopy of inkjet-printed quantum rods^129^. **d**, **e** The photoluminescence spectrum and optical microscopy of thermal evaporated organic fluorescent dyes^52^

Traditional LCDs typically use H-sheet polarizers, the thickness of which exceeds 80 µm^124^. However, positioning polarizers outside the LC cells introduces several challenges, such as depolarization, additional reflections, and parallax effects. Adding an in-cell thin film polarizers offers a solution to these challenges. The In-cell polarizer is put between the substrate glass of the LC cell, as shown in Fig. 8a. which can effectively decouple the depolarization effect of the LC layer and the CF, thus significantly reducing light leakage and enhancing the contrast ratio of the display. Thus, in-cell polarizer in LCDs can lead to a more efficient and thinner design with enhanced resolution. To meet the performance requirements of in-cell thin film polarizers, researchers have used azo-dye materials to make the thin-film polarizer with a thickness of only 200 *nm*. The azo-dye thin-film polarizer exhibits outstanding optical properties, including a dichroic ratio exceeding 95, a single piece transmittance >45.3%, and a polarization efficiency of >99.9%^125–128^.

A photoluminescent CF (PLCF) is suggested to work together with the in-cell polarizer. The idea of using PLCF to replace the conventional CF array is to increase the LCD light efficiency and broaden the color gamut of LCD. In real application, there is a major problem when PLCF is applied to LCD, CCMs depolarize the light. To display correctly with LCD, the PLCF must be put out of polarizer groups and, therefore, out of cell glass. However, the non-collimated LCD light diverges a lot after transmitting the thick cell glass. It leads to the color parallax problem. One solution to eliminate the color parallax is using an in-cell polarizer which means the polarizer is put in between the substrate glass of the LC cell. PLCF will be put directly in between the ICP and cell glass.

In making the in-cell polarizer color-conversion LCD, CCMs such as inkjet-printed quantum rods and thermal evaporated organic fluorescent dyes have been developed for PLCF as an example. Green and red quantum rods (QRs) feature a double shell alloy structure with CdSe/Zn_x_Cd_1−x_S/ZnS and CdSe/CdS/ZnS, respectively. In solution, these QRs exhibit a PLQY over 90%, FWHM of 33 *nm* and 40 *nm* for green and red rods, respectively^129^. The normalized photoluminescence (PL) spectrum of red and green QRs is illustrated in Fig. 8b. The ink-jet printed QR converters is demonstrated in Fig. 8c. In addition, green and red organic fluorescent dyes using N, N0-dimethyl quinacridone (DMQA) and tetraphenyldibenzoperiflanthene (DBP) as dopants were also demonstrated in making the in-cell polarizer color-conversion LCD. The green and red CCM films exhibited PLQYs of 44% and 58%, respectively^52^. The organic CCMs offer the advantage of straightforward patterning processing, facilitated by mask-assisted evaporation. Thin color conversion films can achieve peak blue-light absorption of over 99.9% with a mere 500-nm thickness. The photoluminescence spectrum and optical microscopy of fabricated green and red organic CCM layer are depicted in Fig. 8d, e. For high-resolution patterning of the organic emitters, corrugated ultra-thin silicon nitride shadow masks were employed^130^. These corrugations raise the through-apertures, thereby narrowing the gap between the mask and substrate. This closer alignment enhances the precision of material deposition, which enabled us to create high-resolution CCM arrays. The samples demonstrate a high-resolution PLCF pattern. Remarkably, despite the absence of a black matrix in the PLCF layer, there’s no visible crosstalk phenomenon, attributed to the ultra-thin in-cell polarizer which allows each subpixel to operate independently.

However, there are areas where further enhancements can be made. One such improvement is enhancing the display’s contrast, which could be achieved through backlight collimation. Currently, the system utilizes a diffused LED source, which may not offer optimal contrast. Additionally, precise alignment of the LC and polarizer axis, coupled with careful control of the cell gap and adherence to standard fabrication processes, could further improve the display’s performance. Finally, implementing a black matrix within the PLCF layer could significantly enhance the display’s contrast by eliminating scatter and lateral light propagation (crosstalk), which are common issues in display technologies. These improvements, once addressed, could significantly elevate the performance and quality of high-resolution displays with in-cell polarizers.

### Black matrix

**Fig. 9:**
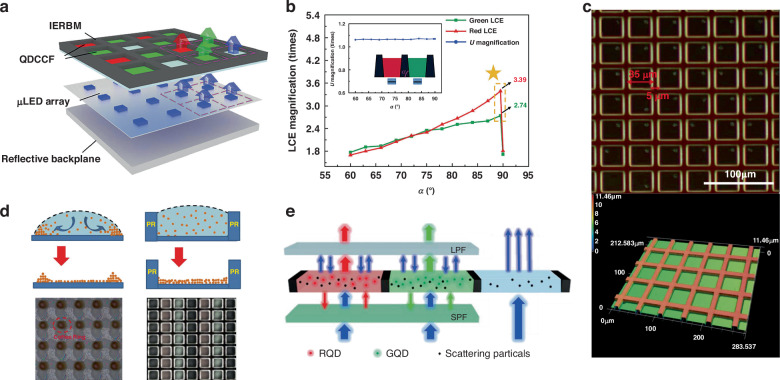
Black matrix and DBR for display effect improvement. **a** Schematic for QD-converted Micro-LED display architecture based on the IERBM^135^. **b** Simulation results of the IERBM based on different tilt angles^135^. **c** PR mold with barrier walls^136^. **d** Schematic illustration of PR mold reducing coffee ring effect^136^. **e** QD CCM with double layers of long pass filter (LPF) and short pass filter (SPF)^137^

The black matrix is essential for the color conversion of micro-sized CCMs. It is used to isolate three-primary-color QD subpixels, thereby preventing color crosstalk and improving the display contrast^131,132^. However, traditional black matrices are made of black photoresist materials that absorb most of the light incident^133,134^. This absorbed light energy accounts for a large portion of LED emission because of the Lambertian intensity distribution of the LED and the isotropic scattering of the CCMs. It is obvious that this absorptive black matrix will greatly affect the light output and reduce the optical conversion efficiency of CCMs. The recovery or reuse of the light energy absorbed by the traditional black matrix has important practical significance for the improvement of the light conversion efficiency of LED displays. Chen et al. proposed a black matrix of internal and external reflective interfaces (IERBM) to achieve higher light conversion efficiency and blue light utilization in QD-LED displays (Fig. 9a). By optimizing the tilt angle and top side length of IERBM, the light conversion efficiency is increased by 3.39 times, and the blue light utilization magnification reaches 1.11 times. At the same time, 1.72% color crosstalk and 89.8% color gamut (Rec.2020) are comparable to traditional absorption black matrices (as shown in Fig. 9b)^135^. Kuo et al. proposed a PR anti-crosstalk window mold, which can not only suppress the optical crosstalk of Micro-LED displays, but also solve the coffee ring effect, which has double improved the display effect^136^. The structure of the window mold is shown in Fig. 9c, which consists of a CCM injection window and a barrier wall to reduce crosstalk. The window size is the same as the sub-pixel size of the Micro-LED array. The barrier wall is silvered to prevent emission and light leakage. Here, without the crosstalk protection mode, there is no clear isolation between each sub-pixel in the CCM Micro-LED array. The blue CCMs overlap with the red and green CCMs, with a degree of crosstalk of about 32.8%. In contrast, the Micro-LED array with an anti-crosstalk mold has almost zero crosstalk, effectively reducing the optical crosstalk between each sub-pixel in the display in the CCMD Micro-LED array. The PR anti-crosstalk window mold addresses the coffee ring effect by controlling liquid thickness homogeneity, preventing CCM particle migration, as depicted in Fig. 9d^136^.

### Distributed Bragg reflectors

To mitigate the issue of excessive blue light leakage in CCM films or pixels, Ye et al. introduced distributed Bragg reflectors (DBR) into color conversion devices. As shown in Fig. 9e, a long-pass filter (LPF) was positioned above the QD CCM layer, while a short-pass filter (SPF) was placed below. The LPF reflects unconverted blue light for secondary excitation, and the SPF redirects backward-emitted red and green light upward. This structure enhances the luminous intensity of red and green pixels by 2.2 times and 2.3 times, respectively, resulting in higher light utilization and color conversion efficiency^137^. In the exploration of the multi-primary-color QD CF, Lin et al. incorporated DBR to achieve the discriminative transmission and reflection of incident light^138^. This integration serves the overarching objective of amplifying the absorption of blue light within the device. The application of DBR in other works of Micro-LED also demonstrates significant improvements in light intensity and blue light suppression^7,46,82,139^.

## Future outlook

For an immersive AR/VR or 3D display experience, resolution stands out as one of the most critical features^140,141^. Higher pixel density contributes to enhanced resolution, offering finer detail and delivering a more realistic image to users. Considering that most AR/VR or 3D display devices are wearable with smaller display sizes (typically 1 ~ 2 inches for smart glasses), achieving high resolution necessitates a small pixel size^142^. Pixel sizes in high-resolution AR/VR or 3D display applications should be reduced to a few µm or less to avoid screen blurring, pixelation, and screen-door effects^143,144^. This is a challenge that current displays based on OLEDs, traditional inorganic LEDs, and LCDs cannot address. In such cases, smaller devices like Micro-LEDs can potentially replace these technologies due to their combination of the advantages of inorganic LEDs with smaller dimensions, reaching the micron or even sub-micron level. However, reducing the device size below the critical size leads to a decrease in the external quantum efficiency (EQE) of traditional quantum well LEDs, creating an efficiency cliff that poses a challenge for the Micro-LED market. Furthermore, supporting a panchromatic architecture by transferring or structuring individual RGB pixels becomes difficult. While the use of RGB Micro-LEDs prepared separately on different substrates is considered, it may not be suitable for full-color micro-display panels due to the limitations in efficiency and the manufacturing process.

Another approach to achieve an RGB full-color Micro-LED display is through color conversion. However, the lack of high-resolution pattern material and process that does not affect the optical properties of the micro transmitter is a major obstacle hindering high-resolution AR/VR or 3D displays. Hence, it is essential to develop appropriate patterning material and processes to support high-resolution displays. Among various patterning techniques, direct optical patterning methods that eschew photoresist, such as inkjet printing, template-assisted printing, and direct laser writing hold great promise in aspects pertaining to uniformity of patterning, process simplicity, and high-fidelity pattern reproduction^4,145^. Future studies on direct optical modes are anticipated to focus on minimizing the loss of optical and electrical properties of the emitters and improving material stability. Simultaneously, the development of advanced inkjet printing or other printing methods capable of achieving high resolution and uniform pattern contours will also hold significant research significance. Additionally, in QD-based micro-displays, brightness should ideally be around 10^5^ *cd.m*^*-*^*²* (preferably, ≈10^6^ *cd.m*^*-*^*²*), considering the significant light loss in displays like AR/VR or 3D displays for outdoor use and optical combinators. However, achieving such high brightness can lead to severe photoinduced degradation based on the QD layer (in down-conversion PL and EL applications). Therefore, the development of QD materials with high quantum yield and high stability becomes crucial.

**Fig. 10:**
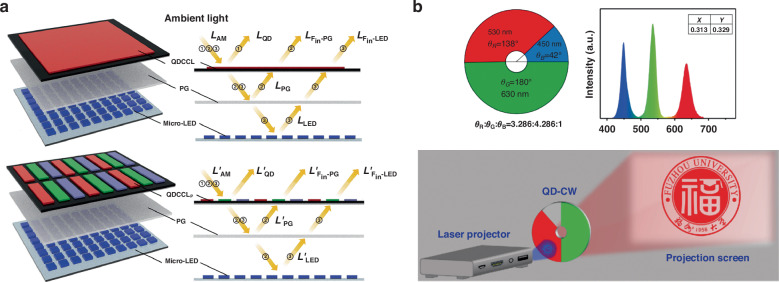
Future research: ambient contrast ratio and projection display. **a** Schematic diagram and the optical path of the color converted micro-LED with a QD CCM layers^146^. **b** Projector prototype based on the QD-CW device^147^

The emergence of Micro-LED technology has heralded a paradigm shift in the display industry, significantly enhancing the dynamic range and outdoor readability of displays. However, the presence of CCMs can also be stimulated by ambient light, resulting in adverse effects on display readability. In the pursuit of broadening the applications of color-conversion displays, this phenomenon becomes a crucial concern. Deng et al. investigated the ambient contrast ratio (ACR) of Micro-LED displays with a QD CCM layer, recognizing the need for a display adaptable to various ambient lighting conditions for an optimal viewing experience. They proposed an ACR calculation model that quantifies screen readability, serving as a reference standard for ACR estimation in emissive color conversion displays (Fig. 10a). The impact of ambient light entering and exciting QDs is non-linear, sharply decreasing at low illuminance and slowing down at high illuminance, potentially reaching a very low ACR level^146^.

In projection displays, the color wheel serves to temporally decompose incident white light into primary colors, distributed across multiple channels on this component. Traditional color wheels with filters prove inefficient. The use of QDs instead of absorptive metal coatings results in a 40% expansion of the color gamut and improved image contrast. Yan et al. investigate QD color wheels (QD-CW) for projection displays (Fig. 10b), demonstrating, through theoretical derivation and simulation, that a color ratio of θ_R_: θ_G_: θ_B_ = 3.286: 4.286:1 achieves standard D65 white balance within one rotation cycle^147^. A QD-color wheel prototype is built for constant-speed rotation, confirming the applicability of QD-CW in projection displays and related domains. In comparison to the currently popular laser projection displays, this strategy of upgrading color wheels offers lower costs, higher flexibility, and a more mature technological foundation for projection. Although currently slightly inferior in color purity compared to laser displays, this approach lays the groundwork for integrating CCM into projection displays, potentially enabling synergistic combinations with laser displays in the future, thereby achieving exceptional display performance.

## Conclusions

Color-conversion displays have become a significant technology in the display industry market today. As shown in Table 3, color-conversion displays offer advantages such as high brightness, wide color gamut, improved contrast ratio, and simplified fabrication processes. These advancements have reinvigorated various display technologies and are driving the introduction of new LCD, OLED, and Micro-LED display products. Currently, color-conversion LCDs have reached maturity in the market. However, there is still a need for advancements in terms of high resolution, low cost, and high reliability. The use of thin film in-cell polarizers can address issues associated with the conventional out-cell polarizer approach, such as depolarization, additional reflections, and parallax effects. This design offers promising prospects for high-resolution and ultra-thin LCDs. The reliability of emerging CCMs, such as MHPs, also needs improvement to drive LCDs toward the wide color gamut required by the Rec.2020 standard. Color conversion OLEDs have already been commercialized by companies like Samsung and Sony. However, scientific research on color conversion OLEDs is limited due to the proprietary nature of the technology, which is predominantly driven by industry rather than public research. The details of this technology are kept confidential as it thrives within the OLED industry. Extensive research is currently focused on color conversion Micro-LED displays, as this approach is believed to be a feasible pathway for commercializing this emerging display technology. The improved optical properties of CCMs present opportunities for their utilization in Micro-LED displays. However, the fabrication process and device design for color conversion Micro-LEDs are still uncertain. Additionally, the stability and protection methods for CCMs on blue LEDs need improvement to meet the strict stability requirements of commercial displays. Therefore, substantial further research into industry-compatible large-scale patterning, proper packing, and encapsulation is crucial to enable the development of commercial products in this field.

**Table 3:** Pros and cons of the color-conversion displays

Pros	Cons
• High brightness: use of intensive blue source; • Wide color gamut: use of high color purity CCMs; • Simplified fabrication processes: only blue light source is needed.	• The device and production technology are not mature; • Potential reliability issue because of the stability of CCMs; • High cost because of the unverified production yield.
